# Liver Transplant Recipients Quality of Life Instrument: Development and Psychometric Testing

**DOI:** 10.5812/hepatmon.9701

**Published:** 2013-10-01

**Authors:** Zohreh Parsa Yekta, Zahra Tayebi, Hooman Shahsavari, Abbas Ebadi, Razieh Tayebi, Fariba Bolourchifard, Forough Rafii

**Affiliations:** 1 School of Nursing and Midwifery, Tehran University of Medical Sciences, Tehran, IR Iran; 2 Department of Nursing and Behavioral Sciences Research Center, Baqiyatallah University of Medical Sciences, Tehran, IR Iran; 3 Depatment of Educational Psychology, Faculty of Psychology, Kharazmi University, Karaj, IR Iran; 4 Centre of Nursing Care Research, School of Nursing and Midwifery, Iran University of Medical Sciences, Tehran, IR Iran

**Keywords:** Liver Transplantation, Quality of Life, Questionnaire

## Abstract

**Background:**

Liver transplantation is a life-saving intervention for many patients with end-stage liver disease. In the past, evaluation of successful liver transplantation was based on patients’ survival rate. However, in recent years this evaluation has been based on patients’ quality of life. Various instruments have been developed to evaluate patients’ quality of life. Nonetheless, scholars still believe that it is crucial to develop a standardized and disease specific instrument for evaluating the quality of life in liver transplant recipients.

**Objectives:**

The aim of this paper was to describe the development and psychometric testing process of a quality of life instrument specific to liver transplant recipients.

**Materials and Methods:**

Initial items of this instrument were extracted from a conventional content analysis study, and then were completed with findings of related international literature. The face validity was assessed by interviewing with four liver transplant recipients, and the content validity was evaluated by eleven experts in the field of transplantation. The construct validity was achieved by involving 250 liver transplant recipients through exploratory factor analysis method, and reliability was calculated by Cronbach's alpha.

**Results:**

Three main factors with 40 items were extracted from the exploratory factor analysis: Health Satisfaction, Concerns, and Complications. Reliability of the instrument was confirmed (alpha = 0.922).

**Conclusions:**

Given the special considerations regarding liver transplant recipients, this questionnaire is more accurate in evaluating the success of liver transplantation.

## 1. Background

Liver transplantation is a life-saving intervention for many patients with end-stage liver disease. In the past, evaluating the outcome of liver transplants was based on patients’ survival rate. Statistical information shows that the survival rate of liver transplant recipients has significantly grown in the past two decades due to developments in the treatment and care, so that now the survival rate in the first and the fifth years after transplantation are 90% and 70% respectively (1, 2).

With recent improvements in patients’ survival rate and graft as the primary indicators of successful liver transplants, health care professionals have been paying more attention to other indicators of a successful liver transplant such as recurrence of underlying disease, transplant’s complications, and recipients’ quality of life (3); in fact, improving patients’ survival rate is not the only purpose of liver transplant procedure. Recovering the patients’ social disability and improving their quality of life are the most expected outcomes of this intervention. The quality of life measure provides a set of useful practical information about the patients’ health status for health care professionals. This measure is an important parameter in assessing diseases’ effects and also evaluating the impact of medical interventions on the overall performance of individuals’ life. Regarding liver transplantation, assessing patients’ quality of life offers information about the desirable and undesirable consequences of related predictive factors (4, 5). Since evaluating patients’ quality of life has an important role in diseases’ managements, various instruments have been developed (6). An accurate estimate of quality of life is strongly dependent on the psychometric properties of developed instrument to measure this construct.

### 1.1. Necessity of Developing a Specific Liver Transplantation Instrument

Many studies have been intended to use quality of life in liver transplant recipients. For this reason, they tried to employ different instruments to measure this construct. Jay and his colleagues conducted a systematic review to evaluate the currently available quality of life instruments used in liver transplants recipients. This study critically appraised the psychometric properties of those instruments and their ability to consider specific concerns of liver transplant population. The authors concluded that none of the current instruments have considered certain key issues of transplant recipients, such as postsurgical complications (e.g. incisional pain, herniated wounds, scars, and deformity caused by operation), anxiety associated with the fear of an unsuccessful transplant, risk of malignancy and opportunistic infections, long term drug side effects, relapse and recurrence of the underlying disease, diabetes and kidney failure. The authors believe that the development of a standard and specific instrument is necessary to understand the factors affecting the quality of life in liver transplant recipients (7).

### 1.2. Liver Transplantation in Iran

Liver transplant has been performed in Iran since 1993. Until 2000, only forty liver transplants had been performed. This number rose to 400 cases between 2000 and 2007, and now more than 1600 liver transplants have been reported. According to the latest studies, the survival rate of recipients in the first and sixth years after transplantation were 84% and 82% respectively (8). Due to the growing number of liver transplant recipients, there is a need to assess their quality of life via a standard and context-based instrument. The importance of context is to the extent that the World Health Organization has defined the quality of life as individuals’ perception of their status in life, cultural and value systems in relation to goals, expectations, standards and concerns. Similar to international context, there is no standard, context-sensitive quality of life instrument specific to liver transplant recipients in Iranian context.

## 2.Objectives

Therefore, the aim of this study was to design and establish a specific instrument for measuring the quality of life in liver transplant recipients.

## 3. Materials and Methods

This study was a methodological research. This method is often used when the aim of the study is to design or validate an instrument (9). To develop the instrument, combinations of inductive and deductive approaches were used. The development process of this instrument was completed through two sequential phases including the item generation and the item reduction over a two year period, from 2010 to 2012.

### 3.1. Item Generation

In this step, the results of a qualitative study with conventional content analysis approach (10) was used to extract the definition and related items of quality of life based on real experiences of liver transplant recipients. In this study, which recruited 9 cases of liver transplant recipients, semi structured interviews were used to collect data. Based on the analysis of these data, the initial items related to quality of life were extracted. After that, these extracted items were verified and completed by international literatures. This review included an extensive literature review on quality of life resources, particularly previous qualitative studies, and also existing quality of life instruments, specifically Ferrans and Powers’ quality of life questionnaire. In addition, throughout the selection of items and designing the initial questionnaire, suggestions and necessary considerations related to shortcomings of current instruments offered by Jay et al. were regarded. Finally, the initial questionnaire (item pool) with 59 items was provided for the second phase of the study. This phase lasted about 6 months.

### 3.2. Item Reduction

In this phase, psychometric properties of initial instrument were evaluated, and necessary modifications were performed. Through this phase, evaluating the instrument’s validity and reliability as well as reducing its items and demonstrating its dimensionality was performed. 

#### 3.2.1. Face Validity

To confirm the face validity, four liver transplant recipients were individually interviewed. Clarity, ambiguity and difficulty of each item were reviewed and discussed. Finally, ambiguous and obscure words and sentences were adjusted.

#### 3.2.2. Content Validity

To ensure the content validity, we used a quantitative approach in the form of the content validity index (CVI) offered by Waltz and Baussel (11). For this reason, eleven experts involved in liver transplant, including nurses, liver transplant coordinators, and specialists of Gastroenterology were asked to determine the item relevancy using a four-point ordinal rating scale (1: irrelevant; 2: somewhat relevant; 3: quite relevant; 4:highly relevant) (12). Based on the Lynn guidelines on acceptable CVI score, 0.70 was seemed as the cut point for determining whether each item should be removed or preserved (13). Moreover, the experts were encouraged to express further comments and suggestions.

#### 3.2.3. Recheck Face Validity

At this stage to ensure the accountability and comprehensibility of the questions, individual interviews were conducted with three liver transplant recipients.

#### 3.2.4. Construct Validity

To ensure the construct validity, exploratory factor analysis was used. Principal Component Analysis and Varimax Rotation Method were used to extract the factors. At first, the questionnaire was completed by 250 liver transplant recipients (14). Convenience sampling was used in this stage due to patients’ country-wide distribution and difficulties to approach them. For that reason, some recipients who referred to transplantation clinic of Namazi Hospital in Shiraz, a center of liver transplantation in Iran, volunteered to fill out the questionnaire. Informed consent was obtained from all recipients for participating in the study. To enter this study, recipients had to be at least 18 years old to ensure they understood various aspects of quality of life. Besides, they were supposed to receive their transplant not less than 6 months before that time, because it is expected that the patient's social rehabilitation would start 6 months after the transplantation (15).

A receptionist at the transplant clinic, who was familiar with the clinical status and patients, was in charge of completing the questionnaires and addressing participants’ potential concerns. In case the patient was illiterate, the receptionist or someone close to the patient completed the questionnaire. Data collection in this phase lasted one year, from September 2011 to September 2012.

#### 3.2.5. Convergent Validity

At this stage, 22 respondents were asked to fill out the developing questionnaire as well as an Iranian version of sf-36 quality of life questionnaire (16).

#### 3.2.6. Reliability

Internal consistency of items was achieved by using Cronbach's Alpha. We used SPSS software (version 13th), Pearson correlation test, exploratory factor analysis and Cronbach's alpha for the analysis.

#### 3.2.7. Ethical Considerations

This research was approved and authorized by the ethics committee of Research Centre at Tehran University of Medical Sciences.

## 4. Results

Through the item generation phase, the initial questionnaire structured with 59 items was formed. In face validity stage, some items were revised and adjusted based on participant’s comments to improve their clarity and comprehensibility. For example, the item of "lack of involvement in deciding the treatment plan" was replaced with the item of “not involving the patients in their treatment plan". During the evaluation of content validity, from initial version of questionnaire nine items with CVI scores of less than 0.70 were omitted (items number 8, 39, 42, 43, 47, 55, 56, 57, and 58) and two items (28 and 29) were merged together due to their conceptual similarities. Therefore, the number of items was reduced to 49 (Table 1). Then, the questionnaire was reviewed with a number of liver transplant recipients to correct minor ambiguities. Moreover, the questionnaire was revised by a Persian Language expert to ensure the accuracy of grammar and text articulacy. 

**Table 1.** Item lists Before the Factor Analysis

List of Terms	Response Category
**How satisfied are you with?**	very satisfied to very unsatisfied
1. Health status	
2. The amount of energy needed for ordinary routine activities	
3. Ability to care for themselves without help from others	
4. Ability to take on the responsibilities of family	
5. Ability to meet financial needs	
6. Ability to do fun activities and sports	
7. Receive emotional support from family	
8. Receive support from special foundations and charities ^a^	
9. Employment status (i.e., the ability to work at the moment)	
10. Marital relationships and sex	
11. Happiness and tranquility in the family	
12. Tranquility of spirit	
13. Relationship and Closeness with God	
14. Appearance	
15. Sense of vitality and exuberance	
16. Achievement level of personal goals	
17. Sense of control over life issues	
18. Being useful to others	
19. How to provide care after discharge and receiving training by the transplant team ^a^	
20. How to Provide psychology and counseling services after transplantation ^a^	
21. How people treat you as an individual who had liver transplant ^a^	
22. Back to normal life after liver transplantation	
23. Life satisfaction in general	
**How worried are you with?**	very worried to very unworried
24. The cost of immunosuppressant drugs	
25. Obtain immunosuppressant drugs	
26. Taking several drugs simultaneously and forever	
27. Possibility of Medical complications	
28. Problems related to marriage and childbearing ^a^	
29. Financial costs generated following liver transplantation	
30. The feeling of being a burden to family and society	
31. Uncertainty about a happy, healthy and desired future	
32. Lack of access to medical members of transplant team when necessary	
33. Lack of available physicians familiar with transplantation issues in home town	
34. Not care about your opinions in the treatment process	
35. Lack of awareness and sufficient information about self-care	
36. Recurrence of the underlying disease	
37. Transplant rejection	
38. Life In exchange for death of another person^a^	
**How problematic for you?**	very problematic to no problem
39. Weakness and fatigue	
40. Unusual increased appetite ^a^	
41. Pain in site of operation ^a^	
42. Scar formation in operation site	
43. Biliary ducts complications	
44.Unusual weight gain	
45. Developed hyperglycemia after Transplantation	
46. Developed hypertension after Transplantation	
47. Difficulty in renal function (creatinine increase in experiments)	
48. Hand tremor ^a^	
49. Osteoporosis (diagnosed by a physician)	

^a^ deleted items in final version (after factor analysis)

In the next step, prepared questionnaire was changed to a Likert scale format, and was given to 250 liver transplant recipients. A total of 250 liver transplant recipients completed the questionnaire. The mean age of participants was 37 years with an average of 3 years post-transplant; 63% of the samples were men. 70% of the participants were married. Most of the subjects (40%) had a high school diploma or above, and approximately 44% of them were employed. Most patients had either hepatitis B or cirrhosis before transplantation. The results are shown in Table 2.

After the descriptive analysis of the data, the appraising of the appropriateness of data for factor analysis was considered. In this way, two main tests including Kaiser-Meyer-Olkin (KMO) Measure of Sampling Adequacy (17) and Bartlett's Test of Sphericity (18) were performed. According to the results of KMO = 0.709 and Bartlett's test = 2628.236 (P <0.001), it could be concluded that using factor analysis regarding sample adequacy and dimensionality was possible.

**Table 2.** Demographic Characteristics of Participants (n = 250)

	Data
**Age, y, Mean ± SD**	37.5 ± 12
**Sex, %**	
Male	63.3
Female	36.7
**Education, %**	
Academic education	19.6
Diploma and under diploma	80.4
**Marital status, %**	
Married	69.7
Single	30.3
**Employment, %**	
Employed	44.6
Unemployed	21.1
Housekeeper	24.4
Student	9.9
**Underlying disease, %**	
Hepatitis	25.2
PSC	12.6
Cirrhosis	42.1
Autoimmune	5
Others	15.1

The results of the analysis were as follows: First, exploratory factor analysis was conducted with a minimum load factor and regardless of the number of factors, 14 factors were extracted at this stage. Since the scree plot showed three or four main factors, further analysis was performed with three and then with four factors. At the end, three-factor analysis seemed to be more practical. These three factors with a cumulative variance of more than 40% covered most of the total observed variance (Table 3). The chosen threshold for factor loads was 0.4. Therefore, nine items including questions 8, 19-21, 28, 38, 40, 41, and 48 were deleted. Only one item was cross load (item 31) and although factor load of this item in the first factor was higher (0.516) than the second factor (0.411), the concept of this item was closer to the second factor and so was located there (Table 3). The results showed that the first factor was accompanied by the maximum loading of questions 1-7, 9-18, 22, 23, 30, and 39 (Table 1). The second factor was accompanied by the maximum loading of questions 24-27, 29, 31-37, and the third factor by questions 42-47, and 49. Therefore, the questionnaire was reduced to 40 items. These three factors were named based on the shared meaning of their related items as “Health satisfaction”, “Concerns”, and “Complications” (Table 4). Convergent validity was evaluated by assessing the consistency of the questionnaire with scores of Iranian version of Sf-36 questionnaire. The consistency score was 0.35, and Cronbach's alpha score of each factor was more than 0.7, and the internal consistency score of all the items was 0.922 in total (Table 4). 

**Table 3.** Results Obtained from Exploratory Factor Analysis

Factor Analysis, Questionnaire Items	Component		
	1	2	3
**q1**	0.666	0.160	0.167
**q2**	0.599	-0.053	0.010
**q3**	0.741	-0.010	-0.048
**q4**	0.721	0.130	-0.034
**q5**	0.555	0.385	-0.220
**q6**	0.751	0.072	-0.046
**q7**	0.481	0.143	0.155
**q8**	0.170	0.371	-0.006
**q9**	0.590	0.182	-0.140
**q10**	0.578	0.245	0.043
**q11**	0.723	0.054	0.204
**q12**	0.711	0.192	0.096
**q13**	0.514	0.021	0.215
**q14**	0.722	0.191	-0.106
**q15**	0.811	0.175	0.055
**q16**	0.736	0.348	-0.148
**q17**	0.810	0.072	0.112
**q18**	0.716	0.002	0.019
**q19**	0.097	0.259	0.312
**q20**	0.241	0.237	0.245
**q21**	0.262	0.172	0.044
**q22**	0.660	0.184	0.107
**q23**	0.606	0.262	0.129
**q24**	0.290	0.673	-0.191
**q25**	0.178	0.701	-0.137
**q26**	0.376	0.584	0.017
**q27**	0.150	0.498	0.127
**q28**	0.339	0.323	0.221
**q29**	0.356	0.675	-0.119
**q30**	0.538	0.370	0.060
**q31**	0.516	0.411	0.189
**q32**	0.098	0.559	0.237
**q33**	0.072	0.464	0.337
**q34**	0.130	0.680	0.281
**q35**	-0.073	0.416	0.325
**q36**	0.127	0.683	0.126
**q37**	0.054	0.535	-0.066
**q38**	-0.081	0.209	0.095
**q39**	0.439	0.267	0.311
**q40**	0.141	0.283	-0.016
**q41**	0.155	-0.003	0.263
**q42**	-0.014	0.265	0.541
**q43**	0.290	0.130	0.456
**q44**	-0.166	-0.016	0.528
**q45**	-0.092	0.085	0.748
**q46**	-0.085	0.138	0.753
**q47**	0.146	-0.038	0.639
**q48**	0.081	-0.198	0.381
**q49**	0.089	0.039	0.600
**Eigenvalue**	12.63	4.216	3.11
**Explained variance, %**	21.118	11.440	8.172

These items were placed in three parts based on identified factors. All of them were evaluated based on a four-point Likert scale, in a way that in every part, 1 shows the least desirable response, and 4 the most desirable one. For example, in the first part that assesses (evaluates) “health satisfaction”, number 1 is “very dissatisfied”, number 2 “fairly dissatisfied”, number 3 “fairly satisfied”, and 4 “very satisfied”. The total score of this questionnaire can be a minimum of 40 to a maximum of 160. The average time to complete this paper-and-pencil questionnaire was approximately 15 minutes.

**Table 4.** Descriptive Statistics and Cronbach’s Alpha of the Liver Transplant Recipients Quality of Life Questionnaire

	No. of items	Mean (SD) ^a^	Skewness	Cronbach’ alpha
**Health Satisfaction**	21	67.113 (11.968)	-0.824	0.92
**Concerns**	12	31.194 (8.223)	0.001	0.84
**Complications**	7	23.613 (4.496)	-1.560	0.76

^a^ Scores range from 21 to 84 for Health Satisfaction, 12 to 48 for Concerns, and 7 to 28 for Complication. Higher scores indicate better quality of life

## 5. Discussion

The final questionnaire consisted of 40 items, and three factors of health satisfaction, concerns and complications. The first factor is health satisfaction which covers most items and defines health according to the World Health Organization definition. This factor covers all aspects of health including physical, mental, spiritual, and social. Considering that the ultimate goal of liver transplantation is returning the patient to active life, satisfaction with all aspects of this factor is necessary. The second factor is concerns. This factor is related to concerns of liver transplant recipients in various areas, particularly follow-up issues and costs. These concerns upset the transplant recipients and influence their life satisfaction level after the transplantation. The third factor that is called complications includes physical problems and possible complications after liver transplantation. Although some of these complications are observed in other solid organ transplant recipients, some of these such as bile duct problems, and recurrence of the underlying disease are specific to liver transplant recipients. Occurrence of these complications has negative impacts on patients’ quality of life. Perhaps the only questionnaire developed for liver transplant recipients is Ferrans and Power. This questionnaire has two parts (satisfaction and importance) with a total of 70 questions which measures quality of life. It evaluates different aspects such as health and activities, psychological and emotional, social/economic, and family relationships. In this questionnaire most items are common among all versions, and in each version a few items are included to suite a particular disease. In the liver transplantation version, there are two specific items related to liver transplantation. These items evaluate the satisfaction and importance of having a liver transplant. Nonetheless, qualitative studies that assessed quality of life in liver transplant recipients, have reported several factors affecting patients’ life. Fear of transplant rejection was addressed as one of the issues which affects the quality of life in the case studies (19, 20). Need for support after discharge, participation in decisions related to care, willingness to participate in social activities, and physical complications such as increased appetite, trembling hands, and high blood pressure were other issues (21-24).Therefore, we saw the necessity and value of designing a questionnaire regarding the issues that liver transplant recipients are dealing with. All of the 40 items of the questionnaire were selected with the intention of briefly addressing specific issues of transplanted patients. Psychometric evaluations resulted in adequate reliability and validity of the questionnaire. An example can be seen in Ferrans & Power reports on the validity and reliability (Cronbach's alpha of 0.73 to 0.99 and correlation of 0.87 in the test-retest interval of two weeks – content, convergent and structure validity). However, in assessing the convergent validity, compared to the Ferrans & Power questionnaire, we faced a mediocre correlation coefficient which was probably due to our insufficient sample size. However, it seems that this questionnaire is more practical, shorter and more specific in comparison with other available questionnaires.

The purpose of this study was to design a valid and reliable instrument for evaluating quality of life in liver transplant recipients. Evidence shows that the available instruments measuring this concept are somewhat lack practicality. Therefore, we tried to design a tool to address the issues and needs ignored in previous questionnaires. A method combining inductive and deductive approaches was used. In the process of psychometric evaluation of the questionnaire, face, content, construct and convergent validity and internal consistency of the questionnaire were assessed. Finally, 40 items of the questionnaire were covered by three factors: health satisfaction, concerns and complication. Four-point Likert scale was used for giving scores to this instrument. Some important variables affecting the patients’ quality of life which were previously neglected are considered in this questionnaire. Variables such as concerns about the possibility of transplant rejection, cost and complication of immunosuppressive drugs, recurrence of the underlying disease, and the need for transplant medical team follow-ups. It is clear that the use of this questionnaire instead of the common tools can be viewed as a more accurate criterion to assess the outcomes of liver transplants. Since solid organ transplant recipients have similar issues, this tool can be used with other transplant recipients, including kidney and kidney –pancreas recipients. However, there is a need to profoundly study this matter.

Using a nonrandom sampling was the main limitation of this study. However, the descriptive statistics indicated that samples are quite diverse in age, sex, marital status, education level, and underlying diseases.
